# The Quarterly Journal of Psycological Medicine and Medical Jurisprudence

**Published:** 1867-09

**Authors:** 


The Quarterly Journal of Psycological Medicine and Medical Jurisprudence. Edited by Wm. A. Hammond, M.D., etc. New York: A. Sampson & Co.

We have just received the first number of this new Quarterly. It is published in excellent style; the type and paper being of the best quality. It contains 160 pages of reading matter; and is devoted to a most important field of medical study and literature. We earnestly hope it will meet with an abundant patronage. Subscription price $5 per annum, or $1.50 for single copies. Published at 60 Duane Street, New York.

